# Transcriptomic analyses reveal biosynthetic genes related to rosmarinic acid in *Dracocephalum tanguticum*

**DOI:** 10.1038/s41598-017-00078-y

**Published:** 2017-03-06

**Authors:** Huie Li, Yaru Fu, Hao Sun, Yanfu Zhang, Xiaozhong Lan

**Affiliations:** 1 Medicinal Plants Research Centre, Tibet Agricultural and Animal Husbandry College, Nyingchi, 860000 China; 2 College of Agriculture, Guizhou University, Guiyang, 550025 China; 3 Institute of Applied Ecology, Chinese Academy of Sciences, Shenyang, 110016 China; 4 TAAHC-SWU Medicinal Plants Joint Research and Development Centre, Tibet Agricultural and Animal Husbandry College, Nyingchi, 860000 China

## Abstract

*Dracocephalum tanguticum* Maxim, a Lamiaceae species endemic to the Qinghai-Tibetan Plateau and adjacent regions, is an important ornamental, medicinal and aromatic herb. In this study, a comprehensive transcriptome of 18 libraries from six organs namely, roots, stems, leaves, sepals, flowers and seeds of *D. tanguticum* were generated. More than 100 Gb of sequence data were obtained and assembled *de novo* into 187,447 transcripts, including 151,463 unigenes, among which the six organs shared 17.7% (26,841). In addition, all unigenes were assigned to 362 pathways, in which ‘biosynthesis of secondary metabolites’ is the second enriched pathway. Furthermore, rosmarinic acid (RA) is one of the multifunctional phenolic bioactive compounds produced in some Lamiaceae species. The six organs of *D. tanguticum* were confirmed to produce RA. A total of 22 predicted biosynthetic genes related to RA from the transcriptome were further isolated. Two of these genes were identified as candidates by evaluating the correlation coefficient between the RA contents and the expression of the predicted biosynthetic genes in the six organs. The new sequence information will improve the knowledge of *D. tanguticum*, as well as provide a reference tool for future studies of biosynthetic genes related to RA in this species.

## Introduction

*Dracocephalum tanguticum* Maxim, a Lamiaceae species endemic to the Qinghai–Tibetan Plateau (QTP) and adjacent regions, is an important ornamental, medicinal and aromatic herb. This plant is one of the important traditional Tibetan medicines which have been used by locals for a long time. However, no study has been reported on *D. tanguticum* because of the plant’s limited distribution. The high-altitude QTP region has unique climate factors, including low oxygen levels, intense ultraviolet light, and large temperature difference between day and night, as well as prolonged daytime during the short growing seasons, which usually commences on April and ends in October for *D. tanguticum*. All of these harsh factors may be beneficial for the biosynthesis of protective secondary metabolites in *D. tanguticum*.

Rosmarinic acid (RA) is a multifunctional phenolic bioactive compound that possesses astringent, antimutagen, antibacterial, antiviral, antifungal, anti-inflammatory and anti-oxidative activities^1–6^. An increasing number of plant sources have been screened for RA content^7–9^, and biotechnological and metabolic engineering approaches for RA production have been studied in previous reports^10–15^. However, plant sources have remained insufficient in meeting the rising demand of RA in recent years.

Previous literature reported that the amino acids, L-phenylalanine and L-tyrosine, are the two precursors of the RA biosynthetic pathway in *Coleus blumei*, a Lamiaceae species^16^. In the phenylalanine-derived pathway of RA biosynthesis, phenylalanine ammonia-lyase (PAL) catalyses the oxidative desamination of phenylalanine, forming t-cinnamic acid, and cinnamic acid; 4-hydroxylase (C4H) introduces the para-hydroxyl group into the aromatic ring of t-cinnamic acid; and 4-hydroxycinnamic acid CoA-ligase (4CL) catalyses the activation of cinnamic acids with coenzyme A. These three enzymes are also commonly shared by biosynthesis of many different secondary metabolites, such as flavonoids, lignans, coumarins, salicylic acid, stilbenes, and so on^17, 18^. In the tyrosine-derived pathway, tyrosine aminotransferase (TAT) catalyses the transamination of tyrosine to 4-hydroxyphenylpyruvic acid, and this product is then converted to 4-hydroxyphenyllactic acid by hydroxyphenylpyruvate (HPPR). RA synthase (RAS) finally catalyses the formation of RA from the two synthesised precursors^9, 15, 19^.

On the basis of the pathway, the genes for RA biosynthesis from several plant species, including the other five Lamiaceae species *C. blumei* ^16^, *Agastache rugosa* ^20^, *Perilla frutescens* ^11^, *Melissa officinalis* ^21^, and *Salvia miltiorrhiza* ^22, 23^, were cloned and even characterised as RA biosynthetic genes. Since RA biosynthetic genes are commonly shared by biosynthesis of many different secondary metabolites, elucidation of the potential pathway for RA biosynthesis genes would aid the required molecular manipulation to improve the quantity of RA in *D. tanguticum*.

High-throughput RNA sequencing (RNA-seq) is a powerful technology recently used to analyse the expression pattern of secondary metabolism genes from different plant samples, especially from non-model plant species^24–27^. However, sequence data of *D. tanguticum* in the public databases are still lacking. In this research, six organs were sequenced through an Illumina sequencing platform to generate transcriptome data of *D. tanguticum*. RA production and the expression levels of the predicted biosynthetic genes related to RA in the six organs were determined, and potential candidate biosynthetic genes related to RA were analysed. This new sequence information will improve the knowledge of *D. tanguticum*, as well as provide a reference tool for future studies of biosynthetic genes related to RA in *D. tanguticum*.

## Results and Discussion

### RA contents in different organs at various stages

**Figure 1:**
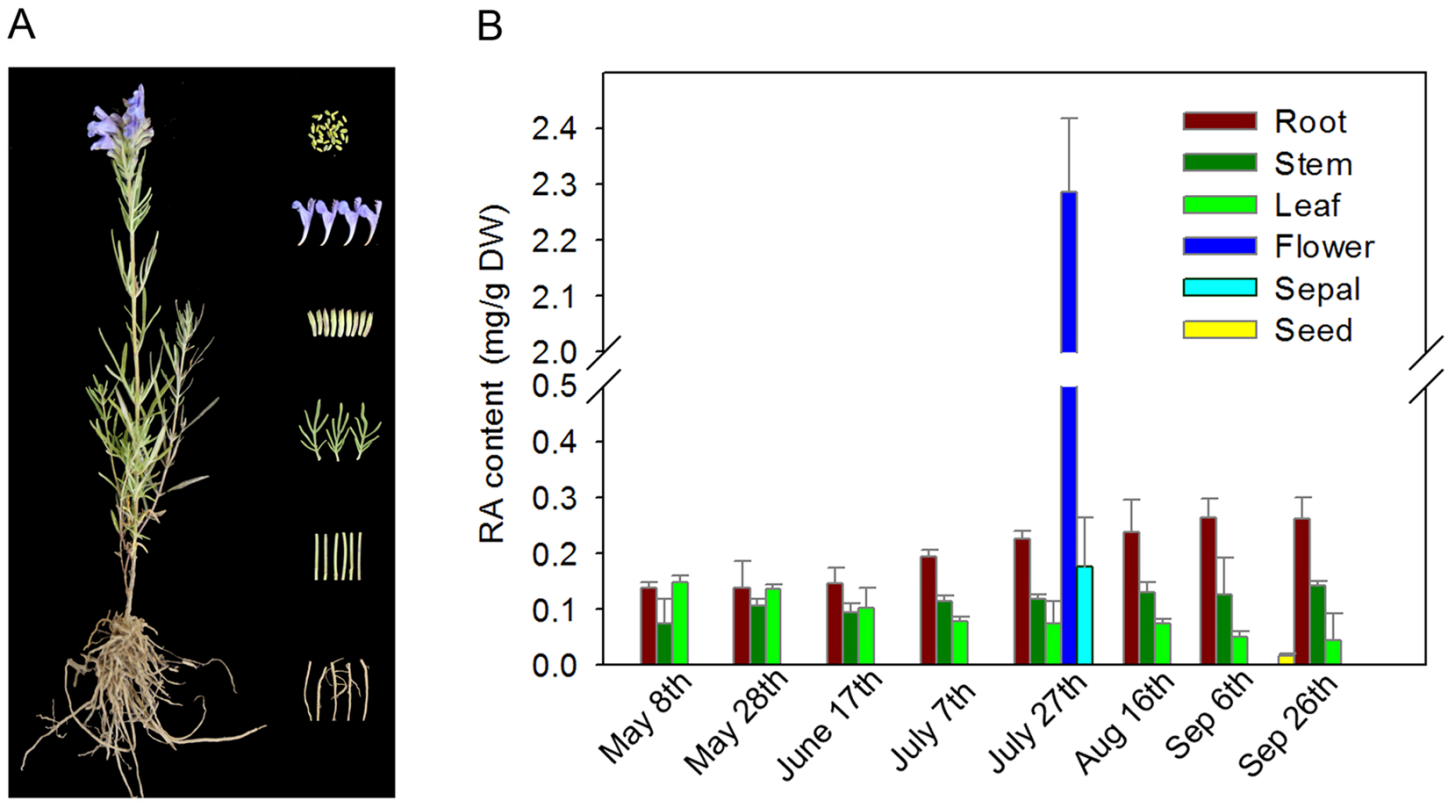
Accumulation of RA in different organs of *Dracocephalum tanguticum* at different stages. (**A**) The whole plant and sampled organs of *Dracocephalum tanguticum*. (**B**) Contents of RA in different organs at different stages. The values and error bars represent the mean and standard error of three biological replicates, respectively.

The six organs, namely, roots, stems, leaves, sepals, flowers and seeds, were sampled (Fig. 1A); the RA contents varied among the different organs at various stages (Fig. 1B). In general, all of the six organs analysed contained RA. The highest content of 2.12 mg/g was observed in the flowers in late July, which is the full-blossom period in the QTP region. The RA contents in the other five organs were below 0.30 mg/g throughout all the growth stages. Among the five organs, the roots exhibited a relatively increasing RA content with the growth season, whereas the leaves showed a decreasing RA content with the growth season. Meanwhile, the RA content in the stems was relatively stable. The lowest content was detected in the seeds in late September. Thus, the five organs, namely, the roots, stems, leaves, flowers and sepals, were sampled in late July, whereas the seeds were sampled in late September for further transcriptome analysis.

Most studies have focused on the RA contents in dried leaves or *in vitro* cultures^15, 28, 29^. In addition, some literature reported RA contents of the different organs of other Lamiaceae species that were consistent with those in our results; the flowers contained the highest RA levels among all the organs tested from *A. rugosa* and *Prunella vulgaris* ^13, 20^.

### Transcriptome sequencing and annotation

**Figure 2:**
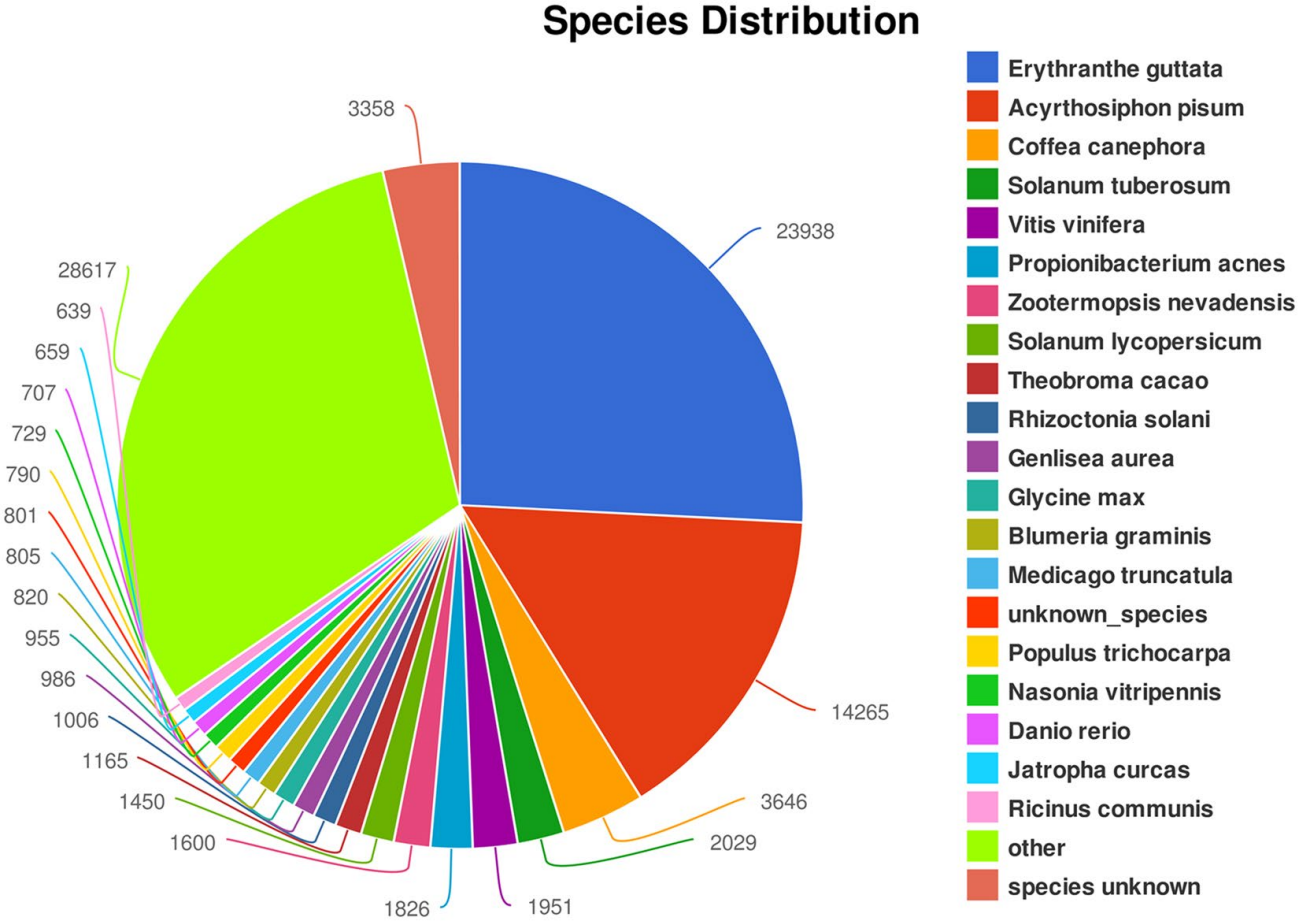
Global transcripts similarity of *D. tanguticum* abundant transcriptome to those of other plant species.

We used Illumina Hiseq technology to establish a transcriptome for *D. tanguticum* and first reported the transcriptome data. The correlation coefficients among the RNA-Seq data of the three triplicates of each organ were all above 0.85 (Table S1), thus verifying the precision of the RNA-Seq data. More than 100 Gb of sequence data from 18 cDNA libraries from the six organs were obtained (NBCI SRA under the accession number PRJNA301920), and *de novo* assembled into 187,447 transcripts, including 151,463 unigenes, from filtered reads. The average lengths of the transcripts and unigenes were 681.79 and 595.47 bp, respectively; half of these lengths (N50) were 1118 and 885 bp, respectively (Table 1). The total raw reads yielded from all the 18 libraries are listed in Supplementary Table S2. Comparison of the *D. tanguticum* abundant transcriptome with the nrNCBI database entries revealed various levels of similarity with the transcriptomes of other plant species (Fig. 2). Among the 187,447 transcripts annotated in the abundant transcriptome, 23,938 transcripts showed high similarity to *Erythranthe guttata*, 3,646 to *Coffea canephora*, 2,029 to *Solanum tuberosum* and 1,951 to *Vitis vinifera*. One of the reasons for such similarity might be the closed genetic relationship to such species. Unlike other highly published plant transcriptomes, the transcripts of *D. tanguticum* were not highly similar to the sequences of model plant species *Arabidopsis*, which may be attributed to the distant genetic relationship between these two species. Interestingly, although the samples were strictly selected and cleaned, the transcripts still showed high similarity to *Acyrthosiphon pisum*, a sap-feeding insect, probably because of the following reasons: (1) contaminant insect secretions were retained within the plant tissues; (2) some gene family proteins of aphids found in various lineages of eukaryotes including plants^30^, for example, the red *A. pisum* individuals could make their own carotenoids, its genome encodes multiple enzymes for carotenoid biosynthesis^31^, suggesting that the species may contain some homologous carotenoid metabolism related genes. These possible reasons, together with relatively small genome (464 Mb) of *A. pisum* in the target database^32^ and the high-throughput sequence data (>100 Gb of sequence data) of *D. tanguticum* in this study, led to high hits; although this insect is common in the fields and may affect the genes expression in this study, the possibility of the homologous gene existing between the two species could not be ruled out. All sequences were then retained to protect the integrity of the transcriptome information.

**Table 1:** Summary of *De novo* transcriptome assembly.

Type	unigene	transcripts
Total sequence num:	151463	187447
Total sequence base:	90191741	127799731
Percent GC:	41.96	41.86
Largest:	14687	14687
Smallest:	201	201
Average:	595.47	681.79
N50:	885	1118
N90:	247	266

The predicted *D. tanguticum* unigenes were classified into three major gene ontology (GO) categories (biological process, cellular component and molecular function) (Supplementary Fig. S1). In terms of cellular component, the top three GO terms were ‘cell part’, ‘intracellular’ and ‘intracellular part’. In terms of biological processes, the most representative categories were ‘organic cyclic compound binding’, ‘heterocyclic compound binding’ and ‘ion binding’. In terms of molecular function, ‘organic substance metabolic process’, ‘primary metabolic process’ and ‘cellular metabolic process’ were the top three GO terms. These findings suggest that the numerous genes involved in the metabolic processes have been detected in *D. tanguticum*.

**Figure 3:**
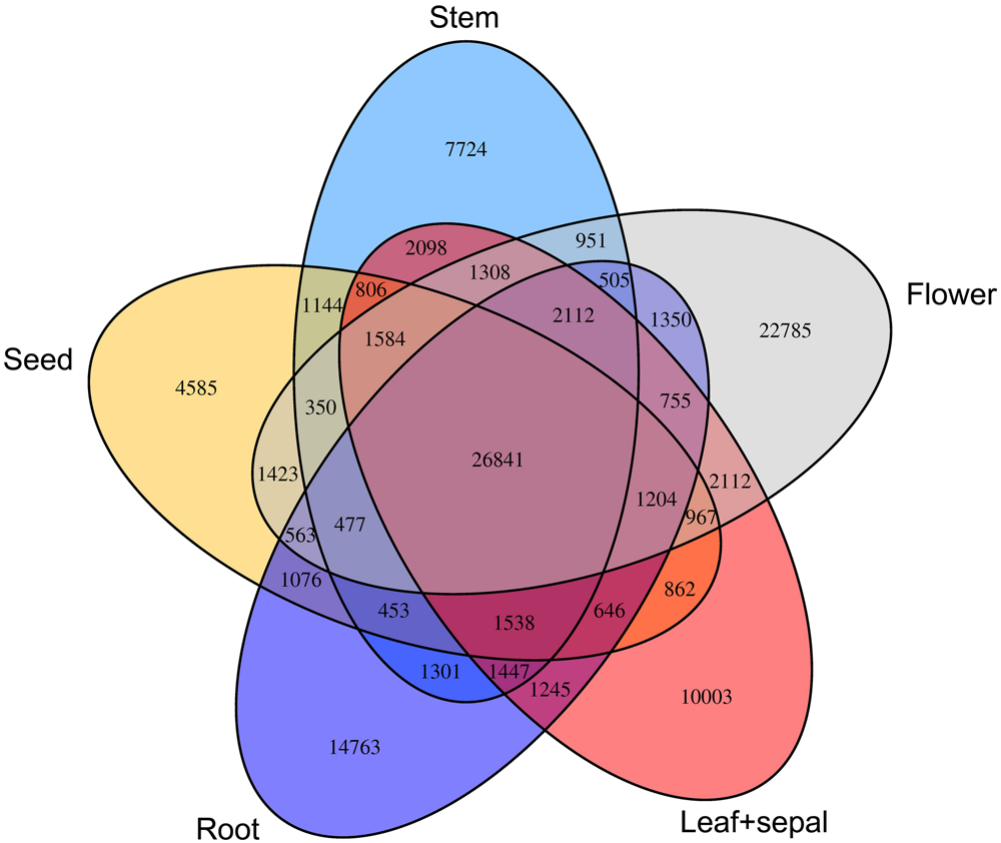
Pathway enrichment analysis in six organs. A significance threshold of *P* < 0.05 was considered statistically significant.

To elucidate which metabolic pathways were enriched, the unigenes were compared against the KEGG database. In total, 86,496 unigenes were assigned to 362 pathways (Supplementary Table S3). The results indicated that ‘metabolic pathway’ (ko01100; 8,544 unigenes) was the most enriched, followed by ‘biosynthesis of secondary metabolites’ (ko01110; 3833 unigenes). Furthermore, 10 molecular pathways were significantly enriched in at least one organ, with one pathways was significantly enriched in five organs (Fig. 3). These 10 pathways were associated with metabolic, indicated that many differing metabolic processes among organs, which including one pathway annotated as ‘phenylpropanoid biosynthesis’ was significantly enriched in five organs; two pathways annotated as ‘Biosynthesis of secondary metabolites’ and ‘Flavonoid biosynthesis’ were significantly enriched in four organs; five pathways, annotated as ‘Flavonoid biosynthesis’, ‘Tyrosine metabolism’, ‘Plant hormone signal transduction’, ‘Photosynthesis’, and ‘Carbon fixation in photosynthetic organisms’, were significantly enriched in three organs; and one pathway annotated as ‘Metabolic pathway’ was significantly enriched in two organs. Plants are well known to biosynthesize secondary metabolites to adapt to environmental stresses^33^. *D. tanguticum* only grows in the QTP region, a high-attitude region with cold, drought, low oxygen, and strong solar radiation environments, thus, it may evolve multifaceted strategies, including synthesis of diverse secondary metabolites, to cope with the diverse stresses. In line with these results, Tibetan wild barley showed diverse and high-content secondary metabolites and their translation levels^34^. In addition, the sampled organs covered the whole plants, which may resulted in numerous predicted unigenes involved in biosynthesis and metabolic processes.

### Identification of simple sequence repeats (SSRs)

SSRs contain high levels of polymorphisms and are widely distributed in plant genomes. The high-throughput sequence data provided a substantial number of SSR candidate markers of *D. tanguticum*. When the length of the SSR fragments exceeded 299 bp and the numbers of repeats for a simple sequence ranged from 5 to 45, a total of 7,623 SSR markers were identified in the transcriptome of *D. tanguticum* (Supplementary Table S4). Among these markers, the most frequent dinucleotide SSR repeat was AG/CT (26.2%), and the most frequent trinucleotide SSR repeat was AAG/CTG (1.7%). These SSR markers identified from the transcriptome would facilitate the further study of the genetic variation in *D. tanguticum* germplasm.

### Comparison of RNA-seq among organs

We clustered the differentially expressed unigenes to understand the differences among the unigenes detected from the six organs (Supplementary Fig. S2). The six organs were divided into two different groups as follows: (1) flowers, roots, and seeds; and (2) stems, sepals, and leaves. The flowers and seeds were grouped together because they are both reproductive organs and shared a greater number of unigenes than other combinations. The second group was composed of three vegetative organs and shared a greater number of unigenes with one another than with other organs. The data of roots were clustered together with reproductive organs probably because this vegetative organ shares more unigenes with the two reproductive organs than the three other vegetative organs. Similar results were also observed in *Allium sativum* ^24^ and *Scutellaria baicalensis* ^35^.

**Figure 4:**
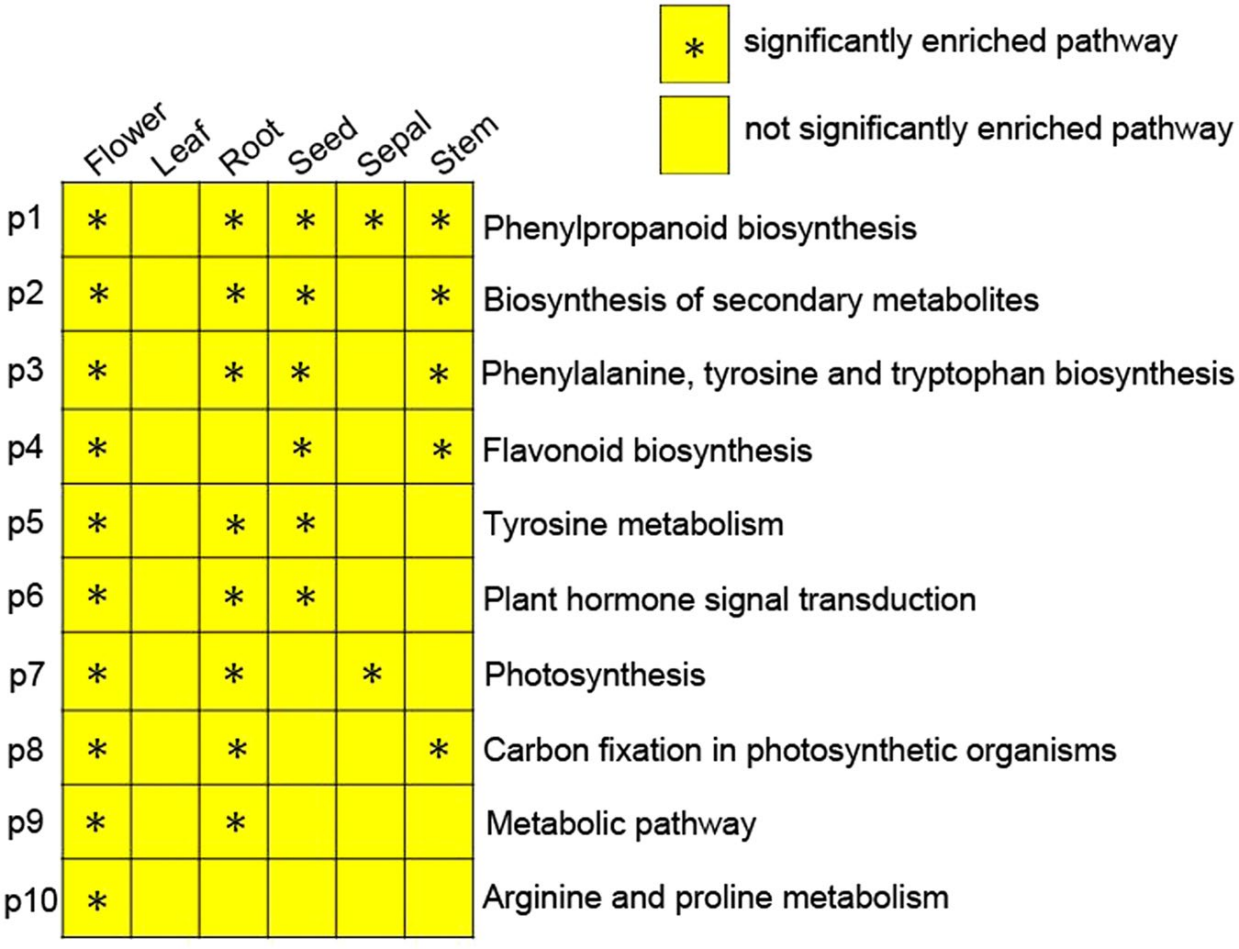
Venn diagram that describes unigenes overlaps among organs of *D. tanguticum*.

The transcripts from the sepals and leaves more closely resembled each other; hence, these two data sets were combined as a single group for further analysis of the same and specific genes among organs. Results showed that these organs shared 17.7% (26,841) unigenes, while the number of specific unigenes was exceptionally high in the flowers (22,785 unigenes) compared with the other five organs (Fig. 4), which represented 15% of the extensive transcriptome. The number of specific unigenes in flowers was followed by that of the roots with 14,763 specific unigenes, the numbers of specific genes for the leaves and sepals, seeds, and stems were 10,003, 4,585 and 7,724, respectively. These data suggested that the organs shared numerous unigenes, and still contain many specific unigenes.

### Prediction and expression of genes encoding enzymes involved in RA biosynthesis

**Figure 5:**
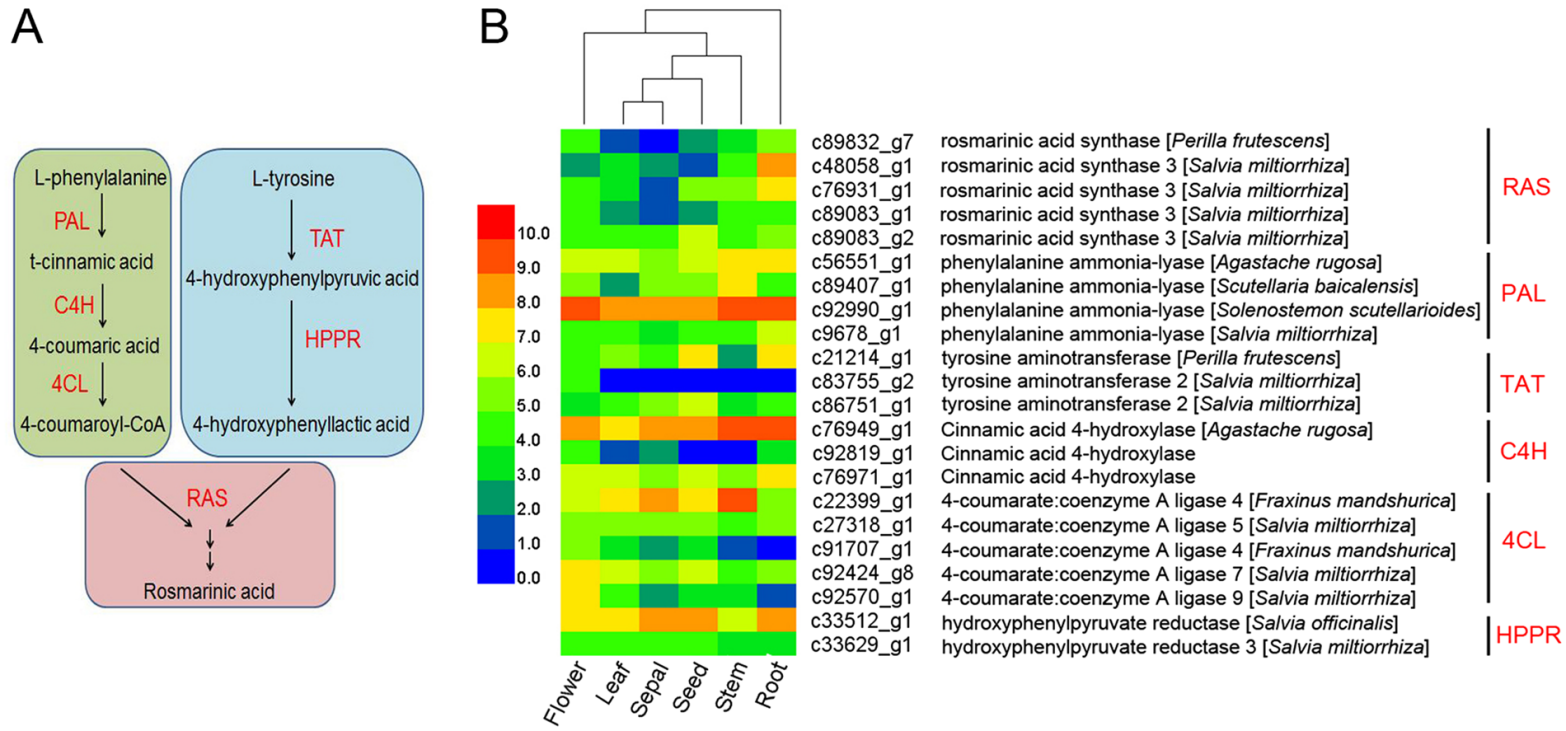
Hierarchical cluster analysis of expression profiles of 22 predicted biosynthetic genes related to RA in the six organs of *Dracocephalum tanguticum*. (**A**) Proposed biosynthetic pathway for RA. (**B**) Heat maps displaying the differential expression of transcripts encoding predicted biosynthetic genes related to RA. Expression values (FPKM) are log2-transformed and then median-centred by variant.

Phenolic compounds play a vital role in the alleviation of abiotic stresses^36^, RA is an important naturally occurring phenolic bioactive compound in *D. tanguticum*. In the present study, a total of 22 unigenes were predicted to encode all the known candidate enzymes^9^ associated with RA biosynthesis in the annotated transcriptome of *D. tanguticum* (Fig. 5), such as four predicted *PAL*s, three predicted *C4Hs*, five predicted *4CLs*, three predicted *TATs*, two predicted *HPPRs*, and five predicted *RASs*. Although RA accumulated the highest content in the flowers among all the organs tested (Fig. 1B), the expression of the predicted genes differed at a greater extent in the roots than in the flowers (Fig. 5). This observation may be attributed to the possible involvement of the predicted genes in other metabolic pathways in the roots.

**Figure 6:**
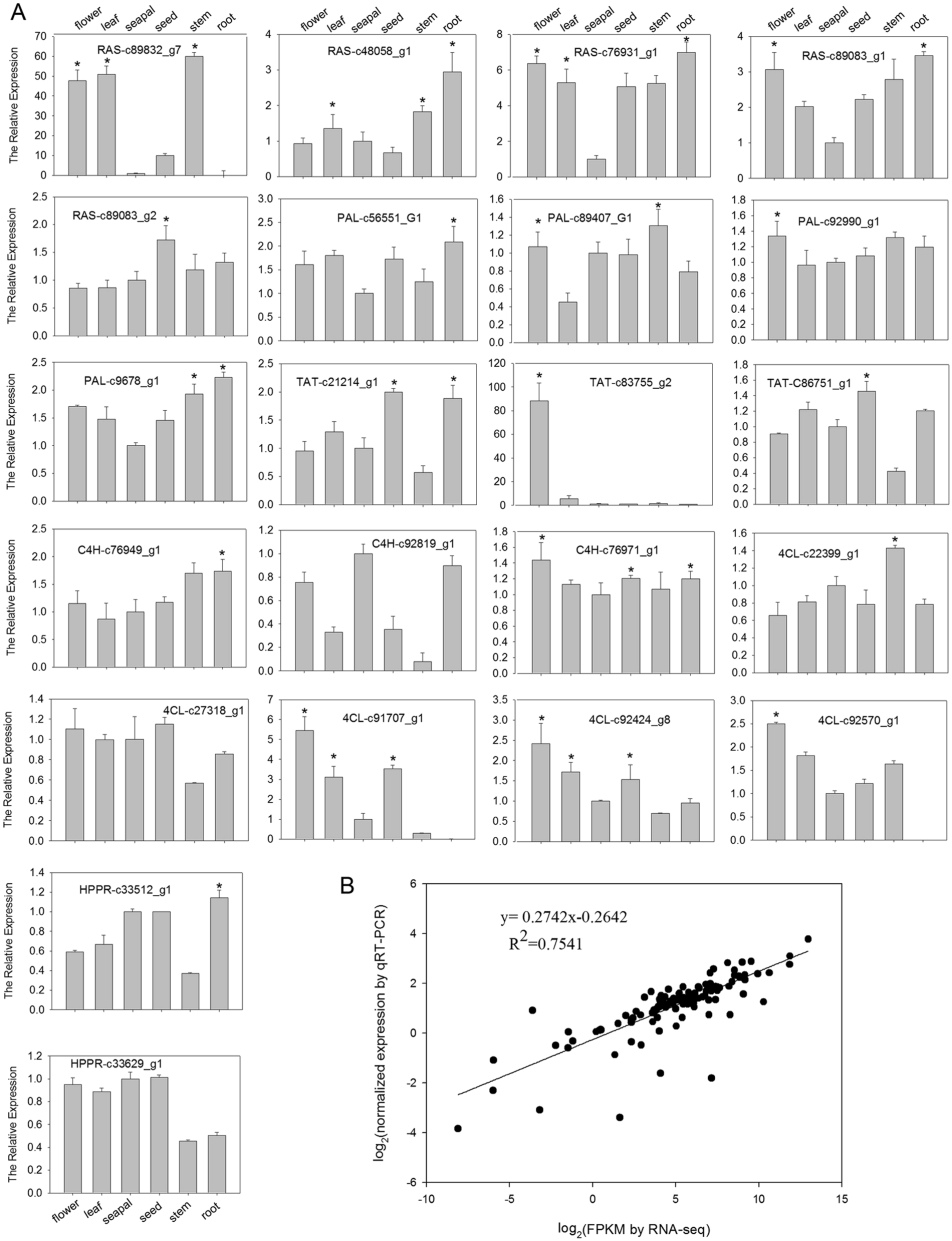
qRT-PCR validation of the expression of the predicted biosynthetic genes related to RA in the six organs by RNA-seq. (**A**) Expression of the genes determined by qRT-PCR. Both *DtACT* and *DtEF1* were selected as the internal controls to normalize the expression. Expression levels of genes in sepals were arbitrarily set to 1 and the other organs were given relative to this. Three biological replicates were performed. A significance threshold of *P* < 0.05 was considered statistically significant. (**B**) Correlation of the expression results obtained from qRT-PCR and RNA-seq for biosynthetic genes related to RA.

To further identify the candidate genes involved in RA biosynthesis, the expression of all the predicted genes in the six organs were determined by real-time quantitative RT-PCR (qRT-PCR) with the *D. tanguticum Actin* (*DtACT*) and *elongation factor 1* (*DtEF1*) as the internal controls. Results showed that most of the expression levels were consistent with the patterns from the transcriptome data in all sampled organs, and the genes from transcriptome expressed at low levels consistently exhibited low signals by qRT-PCR analysis (Fig. 6A). The correlation coefficients of the expression between the 22 predicted genes determined by qRT-PCR analysis and those obtained by RNA-Seq were 0.7541(*P* ≤ 0.05) (Fig. 6B), confirming the reliability of the RNA-Seq data.

### Identification of candidate biosynthetic genes related to RA

**Figure 7:**
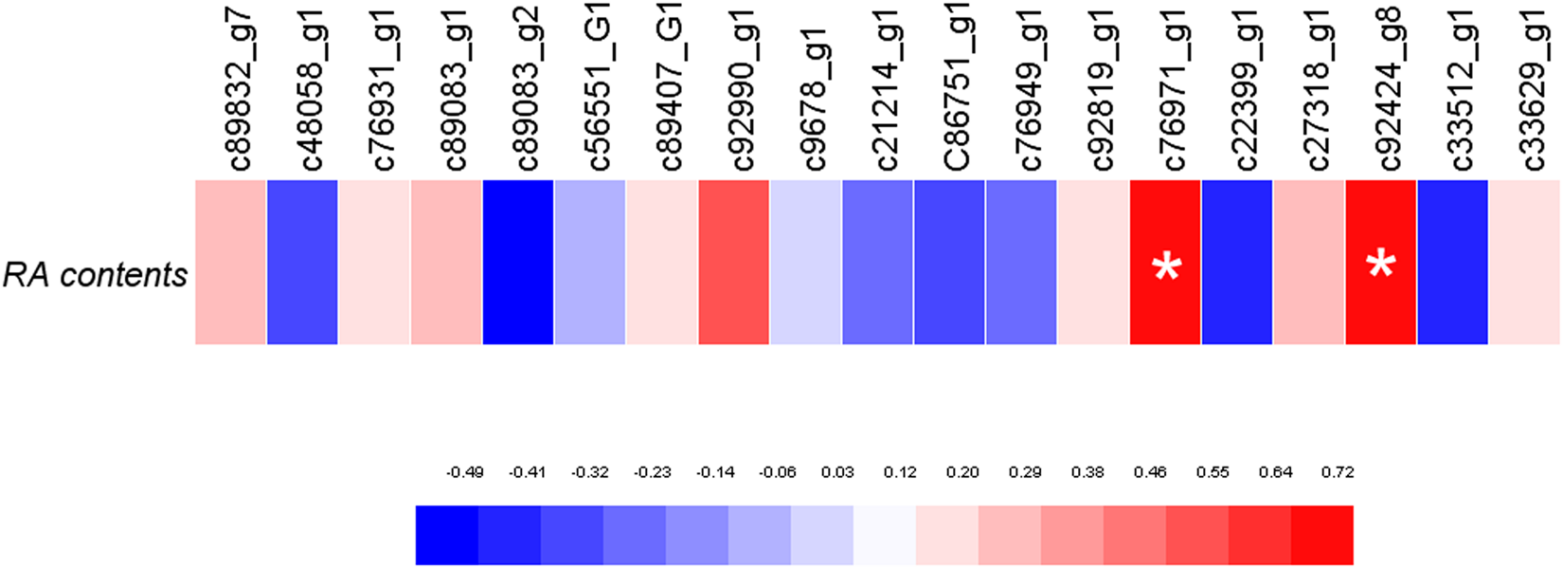
Pearson’s *r* correlation coefficient heat maps showing the association between RA contents and the expression of predicted biosynthetic genes related to RA in the six organs. A significance threshold of *P* < 0.005 was used, which is indicated by ‘*’.

In this study, by analysed the correlation coefficients of the RA contents and the expression of predicted biosynthetic genes related to RA in the six organs of *D. tanguticum*, the RA contents were significantly correlated to the expression of the two predicted biosynthetic genes, which include a *C4H* (c76971_g1) and a *4CL* gene (c92424_g8) (Fig. 7), suggesting that these two genes were involved in RA biosynthesis in *D. tanguticum*. Similarly, RA biosynthesis related genes, such as *SmC4H1*, *Sm4CL3*, *Sm4CL-like1*, and *Sm4CL-like4* in the roots of *Salvia miltiorrhiza*, exhibited high expression in the tissues with high lithospermic acid B, which is a derivative of RA^37^; overexpression of a *S. miltiorrhiza 4CH* in its hairy root cultures increased RA production^23^, and a *S. miltiorrhiza 4CL2* contributed to accumulation of water-soluble phenolic compounds^38^. In addition, these two candidate genes of *D. tanguticum* are all predicted in the PAL pathway of RA biosynthesis, suggests that PAL-derived pathway is more important than TAT-derived pathway in RA biosynthesis of *D. tanguticum*. Further genetic transformation is necessary to confirm the function of the two genes in RA biosynthesis.

## Conclusion

In this study, a comprehensive transcriptome of 18 libraries from the six organs including the roots, stems, leaves, sepals, flowers and seeds of *D. tanguticum* were generated. As a result, more than 100 Gb of sequence data were obtained and assembled *de novo* into 187,447 transcripts, including 151,463 unigenes, the six organs shared 17.7% (26,841) of all. In addition, all unigenes were assigned to 362 pathways, ‘biosynthesis of secondary metabolites’ is the second enriched pathway among them. Furthermore, phenolic compounds play a vital role in the alleviation of abiotic stresses, RA is a multifunctional phenolic bioactive compound produced in some Lamiaceae species. The six organs of *D. tanguticum* and their ability to produce RA were investigated in this study. A total of 22 predicted biosynthetic genes related to RA from the transcriptome were further isolated, and two of these genes were identified as candidates by evaluation of the correlation coefficient between the RA contents and the expression of these predicted biosynthetic genes related to RA in six organs. These new sequences information will improve the knowledge of *D. tanguticum*, as well as provide a reference tool for future studies biosynthetic genes related to RA of the species.

## Materials and Methods

### Plant materials

*D. tanguticum* plants were collected from the Tibet Medicine Plant Germplasm Nursery of Tibet Agricultural and Animal Husbandry College, China. The different organs, including roots, stems and leaves, were harvested from the plants every 20 days throughout the growth season. Flowers and sepals were collected only on July 27, which is the full-bloom stage, while the seeds were collected on September 26, almost the mature period of the seeds. The collected samples were immediately frozen in liquid nitrogen until use.

### Determination of RA contents

RA contents were determined according to the method described by Kim *et al*.^13^ through high-performance liquid chromatography. The determination was conducted with a C18 column (4.6 mm × 150 mm, 5 μm; ZORBAX Eclipse XDB-C18 Analytical, USA) at room temperature. The mobile phase was a gradient mixture of acetonitrile, methanol and 0.2% acetic acid. Afterwards, 5 μL of each sample was injected and analysed with a flow rate of 1.0 mL min^−1^ under a 280 nm wavelength. The RA content of each sample was then calculated using a standard curve. Mean values were obtained from three independent replicates.

### RNA isolation

An EASYspin Plus Plant RNA Isolation Kit (Aidlab, China) was used to isolate total RNA following the manufacturer’s instructions. DNase I (TaKara, Japan) was employed to remove genomic DNA. A 2100 Bioanalyser (Agilent, US) was used to assess RNA quality, and RNA quantity was determined with a NanoDrop–2000 (Thermo, USA). The sequencing library was constructed only with high-quality RNA samples (OD 260/280–1.8 to 2.2, OD 260/230 ≥ 2.0, RIN ≥ 7, 28S:18S ≥ 1.0, >10 μg).

### Library preparation and Illumina Hiseq4000 Sequencing

A TruSeq^TM^ RNA Sample Preparation Kit from Illumina (San Diego, USA) was used to prepare the RNA-seq transcriptome library with 5 μg of total RNA. Messenger RNA was then isolated through the polyA selection method with oligo(dT) beads. The RNA was then fragmented (100–400 bp) with a fragmentation buffer, and double-stranded cDNA was synthesised with a SuperScript double-stranded cDNA Synthesis Kit (Invitrogen, USA) using random hexamer primers (Illumina). The synthesised cDNA was subjected to end-repair, phosphorylation and ‘A’ base addition using the Illumina library construction protocol. Size selection of the cDNA target fragments that were 200–300 bp long on 2% low-range ultra-agarose was performed in the libraries. PCR amplification was carried out using Phusion DNA polymerase (NEB, USA) for 15 PCR cycles. The resultant paired-end RNA-seq sequencing library was then sequenced with an Illumina HiSeq 4000 (2 × 151 bp read length) after the RNA was quantified with TBS380. Three replicates were performed for each organ.

### *De novo* assembly and annotation

The raw paired-end reads were trimmed and quality control was performed with SeqPrep (https://github.com/jstjohn/SeqPres) and Sickle (https://github.com/najoshi/sickle) using the default parameters. RNA *de novo* assembly was achieved with Trinity (http://trinityrnaseq.sourceforge.net/)^39^, which was adopted to clean the data from the 18 libraries. The NCBI protein nonredundant (NR), String and Kyoto Encyclopedia of Genes and Genomes (KEGG) databases were searched with BLASTX using all the assembled transcripts to identify the proteins that achieved the highest sequence similarity with the given transcripts. Their functional annotations were retrieved, and a typical cut-off *E*-value of less than 1.0 × 10^−5^ was set. GO annotations of the unique assembled transcripts for describing biological processes, cellular components and molecular functions were obtained with the BLAST2GO (http://www.blast2go.com/b2ghome) program^40^. The KEGG database (http://www.genome.jp/kegg/)^41^ was used for metabolic pathway analysis. Microsatellites were searched for the unique assembled transcripts with Msatcommander (http://code.google.com/p/msatcommander/)^42^ using the default parameters.

### Differential expression analysis and functional enrichment

The expression levels of each transcript were calculated by the fragments per kilobase of exon per million mapped reads (FRKM) method to identify the differentially expressed genes (DEGs) between two different samples. Differential expression analysis was performed with the R Statistical Package Software EdgeR (Empirical analysis of Digital Gene Expression in R, http://www.bioconductor.org/packages/2.12/bioc/html/edgeR.html)^43^.

Moreover, functional enrichment analysis with GO and KEGG was performed to identify which DEGs were significantly enriched in the GO terms and metabolic pathways. A Bonferroni-corrected *P* value of ≤0.05 was used for comparison with the whole-transcriptome background. KEGG pathway and GO functional enrichment analyses were performed with Goatools (https://github.com/tanghaibao/Goatools) and KOBAS (http://kobas.cbi.pku.edu.cn/home.do)^44^. The significantly enriched pathways among organs were analyzed according to Wang *et al.* ^45^.

### qRT-PCR analysis

Total RNA (1 mg) from each sample was used to synthesise cDNA using the PrimeScript RT Reagent Kit (Takara, Japan), and qRT-PCR was conducted using SYBR premix Ex Taq (Takara, Japan). The analyses were carried out following the procedures described by Li *et al*.^46^ with a modified annealing temperature of 62 °C. The expression levels of *DtACT* and *DtEF1* were most stable according to the comparison of transcript expression among the six organs, thus, the expression of these two genes were selected as the internal controls to normalise the expression of the detected predicted genes. The specific primer pairs adopted are listed in Supplementary Table S5. Three biological replicates were performed.

### Data analysis

One-way ANOVA analysis has been performed using SPSS Software (version 22.0; IBM, NY, USA) according to Ji *et al*.^47^. Pearson’s correlation coefficient evaluations were performed using GraphPad Prism Software (version 6.0; GraphPad, SD, USA). Graphs were generated using Sigmaplot (Version 10.0; Systat, CA, USA) and HEMI (version 1.0, Illustrator, Wuhan, CHINA).

## Electronic supplementary material

Supplementary Files

Table S3

Table S4
