# Supplementary material for: Transcriptomic analyses reveal biosynthetic genes related to rosmarinic acid in Dracocephalum tanguticum
Source: Sci Rep. 2017 Mar 6;7:74. doi: 10.1038/s41598-017-00078-y (PMC5428373; doi:10.1038/s41598-017-00078-y)
Supplement: Supplementary file 1 — Supplementary Files [file 41598_2017_78_MOESM1_ESM.pdf]

Transcriptomic analyses reveal biosynthetic genes related to rosmarinic acid in *Dracocephalum tanguticum*

Huie Li[1,3], Yaru Fu[1], Hao Sun[4], Yanfu Zhang[1], Xiaozhong Lan[1,2]*

[1]Medicinal Plants Research Centre, Tibet Agricultural and Animal Husbandry College, Nyingchi 860000, China. [2]TAAHC-SWU Medicinal Plants Joint Research and Development Centre, Tibet Agricultural and Animal Husbandry College, Nyingchi 860000, China. [3]College of Agriculture, Guizhou University, Guiyang 550025, China. [4]Institute of Applied Ecology, Chinese Academy of Sciences, Shenyang 110016, China. Correspondence and requests for materials should be addressed to X. L. (email: lanxiaozhong@163.com)

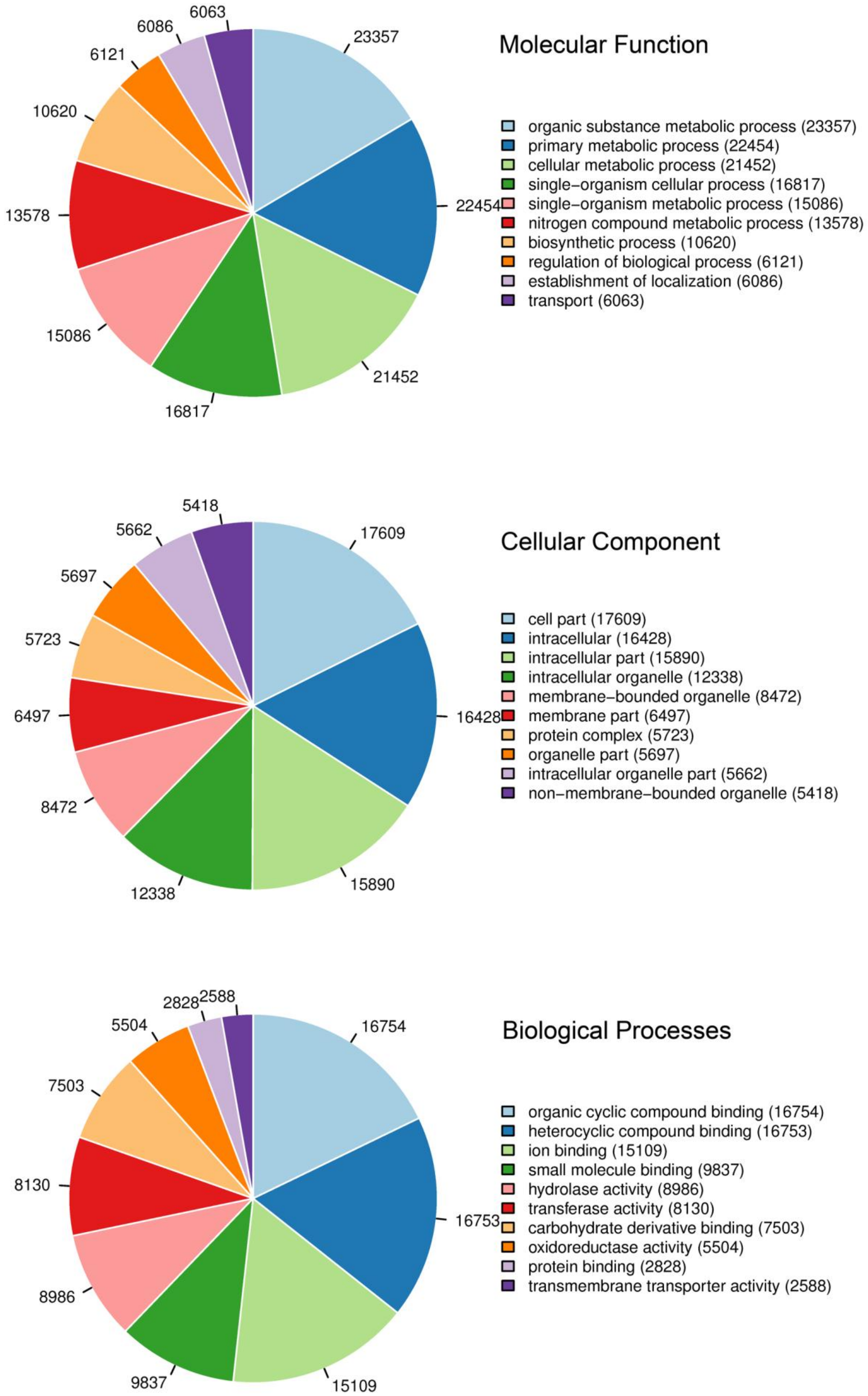

Fig. S1 Gene ontology classification of unigenes.

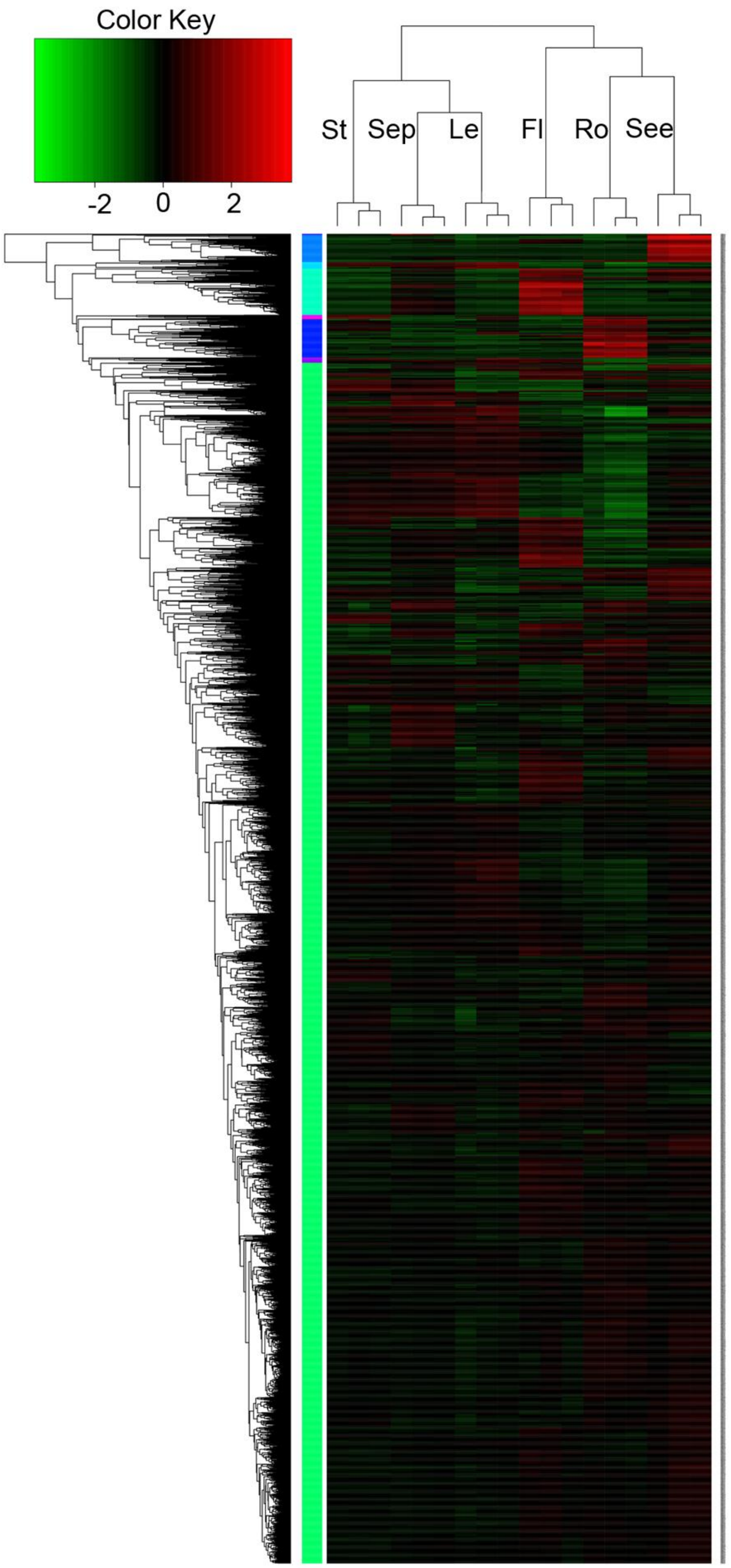

Fig. S2 Clustered the differentially expressed unigenes in the six organs

Table S1 Correlation coefficient of RNA-Seq data among three triplicates of each organ.

Samples	Flower1	Flower2	Leaf1	Leaf2	Root1	Root2	Seed1	Seed2	Sepal1	Sepal2	Stem1	Stem2
Flower2	0.9372											
Flower3	0.9376	0.8956										
Leaf2			0.8823									
Leaf3			0.9367	0.9842								
Root2					0.8589							
Root3					0.8836	0.9128						
Seed2							0.9886					
Seed3							0.9455	0.9355				
Sepal2									0.8672			
Sepal3									0.9847	0.8806		
Stem2											0.9397	
Stem3											0.9821	0.9599

Table S2 Total raw reads yielded from all 18 libraries.

Sample ID	Total Reads	Total Bases	Total Reads with_Ns	NReads%	A%	T%	C%	G%	N%	Error%	Q20%	Q30%	GC%	Adapter %	Read1 Dup(%)	Read2 Dup(%)	Paired Dup(%)
F_1	38445900	5.81E+09	190607	0.5	26.55	25.32	23.59	24.52	0.02	0.0163	95.46	89.42	48.1	41.76	21.54	14.32	4.47
F_2	45308450	6.84E+09	225848	0.5	26.21	25.25	23.85	24.67	0.02	0.0177	94.78	88.15	48.52	38.8	22.54	13.17	3.72
F_3	35235570	5.32E+09	175033	0.5	26.77	25.37	23.4	24.44	0.02	0.0177	94.66	88.18	47.84	42.76	21.51	13.51	4.16
L_1	41033628	6.2E+09	204363	0.5	26.56	25.25	23.64	24.53	0.02	0.0171	95.01	88.67	48.17	41.45	20.8	13.58	4.35
L_2	37761670	5.7E+09	358412	0.95	26.01	24.93	24.12	24.92	0.02	0.0176	94.9	88.23	49.04	39.74	24.95	16.68	5.37
L_3	43359034	6.55E+09	412417	0.95	26.22	25.09	23.86	24.81	0.02	0.0161	95.57	89.51	48.67	40.87	23.19	16.88	5.41
R_1	46047834	6.95E+09	229608	0.5	26.04	24.82	24.08	25.04	0.02	0.0161	95.45	89.46	49.12	43.44	32.72	23.3	11.46
R_2	37916320	5.73E+09	188085	0.5	26.7	25.23	23.5	24.56	0.02	0.0182	94.46	87.78	48.06	44.73	20.91	12.54	4.15
R_3	40672476	6.14E+09	384963	0.95	26.31	25.27	23.77	24.63	0.02	0.0167	95.25	88.96	48.4	38.97	22.56	15.86	4.51
Se_1	32900568	4.97E+09	312878	0.95	25.55	24.17	24.67	25.59	0.02	0.0179	94.7	88.04	50.27	41.77	27.03	19.17	6.76
Se_2	35599046	5.38E+09	337956	0.95	25.73	24.22	24.5	25.53	0.02	0.0173	94.89	88.45	50.03	45.39	26.85	19.00	7.5
Se_3	38669318	5.84E+09	366524	0.95	25.7	24.67	24.36	25.25	0.02	0.0178	94.83	88.12	49.61	40.72	22.05	14.5	4.2
Sep_1	45497520	6.87E+09	431392	0.95	26.59	25.3	23.56	24.53	0.02	0.0162	95.38	89.32	48.09	43.24	20.25	14.56	5.05
Sep_2	40124352	6.06E+09	381825	0.95	26.58	25.42	23.56	24.42	0.02	0.0159	95.57	89.6	47.98	41.02	20.98	15.65	5.19
Sep_3	52795950	7.97E+09	288854	0.55	26.44	25.09	23.74	24.69	0.03	0.0177	95.05	88.28	48.43	41.55	22.88	18.53	6.93
St_1	43063922	6.5E+09	408485	0.95	26.24	25.4	23.84	24.5	0.02	0.0169	95.29	88.85	48.34	34.75	23.41	15.73	3.97
St_2	34987930	5.28E+09	332643	0.95	26.42	25.42	23.64	24.51	0.02	0.0172	95.18	88.64	48.14	39.99	17.59	11.66	2.76
St_3	41809924	6.31E+09	395541	0.95	26.24	25.2	23.86	24.68	0.02	0.0168	95.32	88.93	48.54	40.23	19.77	13.55	3.51

Table S5 Specific primers of genes detected by qRT-PCR.

Gene Family name	Unigene	Primers	Length of PCR products (bp)
RAS	c89832_g7F	ATGCTCTTGTCAGTCACTAGGA	143
	c89832_g7R	CATTCGTCTGAAAAGGGTTTCG	
	c48058_g1F	GTGTGTGTGTGGAGTTCGAAC	136
	c48058_g1R	GTGGGTACATCCACATCAACT	
	c76931_g1F	CTTCGCTGAGCATCACACATC	164
	c76931_g1R	GTGTTCGATGTGGGGGAACT	
	c89083_g1F	GTATGCTACTATTTGACAAGTTGC	158
	c89083_g1R	TGTCATCCCTACTTATATGGAAC	
	c89083_g2F	ATGAGCTTTTCACCGACTTCGT	145
	c89083_g2R	CATCAAACCACGAACAGACAACA	
PAL	c56551_G1F	GGCGGTGGAGCTGGCGGA	192
	c56551_G1R	CCGAATATTCCGGCGTTCAAG	
	c89407_G1F	TCCACATCATTCCACGTGTCG	177
	c89407_G1R	GGAAGTTTTGCAAAGCTTGTGG	
	c92990_g1F	CCCACCAACCACCACAACC	144
	c92990_g1R	GGGGTTTGTTGAGGGTGGATA	
	c9678_g1F	TGAACCTCTACCTATCTGCTAG	159
	c9678_g1R	CAAAGTACATAGGGAGAACTCACA	
TAT	c21214_g1F	AGCCACAGCCAAGATGGATAGT	158
	c21214_g1R	GGGACGGGAGAAAGTTTAGGC	
	c83755_g2F	GGCACTGCACGCCAAGAAAC	162
	c83755_g2R	TATCACACAACTTCCCTCTGAA	
	c86751_g1F	AACTGGTTACGCTTGACATTTGC	138
	c86751_g1R	CAGCGATCATGTCCGAACGG	
C4H	c76949_g1F	GAGAGATGATTCTATGCTGTTAC	164
	c76949_g1R	CGCATCTAATGCTGCAGTTTG	
	c92819_g1F	CGGGCAGTTTGCTACTCGG	205
	c92819_g1R	CACAGCAGATAACGGCTACTTGAA	
	c76971_g1F	GAGGAAAGATCTGAGAGCCAA	191
	c76971_g1R	CGATGAACAGTTTGCTTATCGC	
4CL	c22399_g1F	CAGTACTATCGATGTGTCTGG	150
	c22399_g1R	TGCAGATCTCGCCCGGTTG	
	c27318_g1F	GGGGATTTATGATGTCTGCCT	156
	c27318_g1R	CGCGTGTGTGTGTGTGATGC	
	c91707_g1F	CACCAACGCACAAAGCAGGT	138
	c91707_g1R	AAGAGTCATCCTCAGTGGATG	
	c92424_g8F	GGTGTGAACACCAAGTTTGAAC	202
	c92424_g8R	TAAGTCGTTACGGCCCTATGA	
	c92570_g1F	GCTAGAAGCAGTGTTTCAGAAG	164

	c92570_g1R	GAGCAACCACAACACTACATG	
HPPR	c33512_g1F	GACTTTACACTACTGGAATACTG	175
	c33512_g1R	CTTTGCCCTATCAACCACTGC	
	c33629_g1F	TGCCACGATTGATCAGGTATG	182
	c33629_g1R	CAATAGTTTCACATGGCACAAG	
house keeping	*DtACT*-F	TCGTGAACATGGCGTCGTAC	206
	DtACT-R	CTGCTCTACATAAACTTCTACC	
	DtEF1-F	GGCTGCTGCCAAGAAGGG	194
	DtEF1-R	CCTACCTCGCCACAACCCA	
